# Gastrointestinal disseminated histoplasmosis in HIV-infected patients: A descriptive and comparative study

**DOI:** 10.1371/journal.pntd.0009050

**Published:** 2021-01-22

**Authors:** Mathieu Nacher, Audrey Valdes, Antoine Adenis, Romain Blaizot, Philippe Abboud, Magalie Demar, Félix Djossou, Loïc Epelboin, Caroline Misslin, Balthazar Ntab, Dominique Louvel, Kinan Drak Alsibai, Pierre Couppié

**Affiliations:** 1 CIC INSERM 1424, Centre hospitalier Andree Rosemon Cayenne, Cayenne, French Guiana; 2 DFR Santé, Université de Guyane, Cayenne, Cayenne, French Guiana; 3 Equipe Opérationnelle d’hygiène hospitalière, Centre hospitalier Andree Rosemon Cayenne, Cayenne, French Guiana; 4 Department of dermatology, Centre hospitalier Andree Rosemon Cayenne, Cayenne, French Guiana; 5 Service des Maladies Infectieuses et Tropicales, Centre hospitalier Andree Rosemon Cayenne, Cayenne, French Guiana; 6 Laboratory, Centre hospitalier Andree Rosemon Cayenne, Cayenne, French Guiana; 7 UMR Tropical Biome and Immunopathology, Université de Guyane, Cayenne, French Guiana; 8 Service de Médecine, Centre hospitalier de l’Ouest Guyanais, Saint Laurent du Maroni, French Guiana; 9 Département d’Information Médicale, Centre hospitalier de l’Ouest Guyanais, Saint Laurent du Maroni, French Guiana; 10 Service de Médecine B, Gastroentérologie, Centre Hospitalier Andrée Rosemon, Cayenne, French Guiana; 11 Service d’Anatomopathologie, Centre Hospitalier Andrée Rosemon, Cayenne, French Guiana; Yale University School of Medicine, UNITED STATES

## Abstract

Disseminated histoplasmosis is one the main AIDS-defining opportunistic infections in HIV-infected patients, notably in Latin America. The non-specific and proteiform clinical presentation leads to diagnostic delays that may lead to fatal outcomes. This retrospective multicentric study aimed to describe the frequency and manifestations of gastrointestinal histoplasmosis in French Guiana, and to compare patients with disseminated histoplasmosis with or without gastrointestinal involvement.

Between January 1, 1981 and October 1, 2014 co-infections with HIV and histoplasmosis were enrolled. Inclusion criteria were: age >18 years, confirmed HIV infection; first proven episode of histoplasmosis.

Among 349 cases of disseminated histoplasmosis, 245 (70%) had a gastrointestinal presentation. Half of patients with gastrointestinal signs had abdominal pain or diarrhea, mostly watery. Half of patients with abdominal pain had diarrhea (63/124) and half of those with diarrhea (63/123) had abdominal pain. A significant proportion of patients also had hepatomegaly and, to a lesser degree, splenomegaly. After adjusting for potential confounding, the presence of lymphadenopathies >2cm (AOR = 0.2, IC95 = 0.04–0.7, P = 0.01), Haitian origin (AOR = 0.04, IC95 = 0.004–0.4, P = 0.006) were associated with a lower prevalence of gastrointestinal signs and positive gastrointestinal presence of *H*. *capsulatum*. Persons with a gastrointestinal *H*. *capsulatum* were more likely to have a decreased prothrombin time, lower ferritin, lower liver enzymes, and lower concentrations of LDH than those without gastrointestinal signs and symptoms. They also had a shorter interval between symptoms onset and diagnosis. Patients with a positive gastrointestinal identification of *H*. *capsulatum* were less likely to die at 1 month than those without a gastrointestinal presentation (respectively, 4.6% vs 18.5%, P = 0.01).

Subacute or chronic gastrointestinal presentations are very frequent during disseminated histoplasmosis, they seem less severe, and should lead to suspect the diagnosis in endemic areas. There were populational or geographic differences in the frequency of gastrointestinal manifestations that could not be explained.

## Author summary

This retrospective multicentric study aimed to describe the frequency and manifestations of gastrointestinal histoplasmosis in French Guiana, and to compare patients with disseminated histoplasmosis with or without gastrointestinal involvement. Between January 1, 1981 and October 1, 2014 co-infections with HIV and histoplasmosis were enrolled. Inclusion criteria were: age >18 years, confirmed HIV infection; first proven episode of histoplasmosis. Among 349 cases of disseminated histoplasmosis, 245 (70%) had a gastrointestinal presentation. Half of patients with gastrointestinal signs had abdominal pain or diarrhea, mostly watery. Half of patients with abdominal pain had diarrhea (63/124) and half of those with diarrhea (63/123) had abdominal pain. A significant proportion of patients also had hepatomegaly and, to a lesser degree, splenomegaly. After adjusting for potential confounding, the presence of lymphadenopathies >2cm, Haitian origin were associated with a lower prevalence of gastrointestinal signs and presence of *H*. *capsulatum*. Persons with a gastrointestinal presentation were more likely to have a decreased prothrombin time, lower liver enzyme concentration, ferritin, and lower concentrations of LDH than those without gastrointestinal signs and symptoms. The delay between symptom’s onset and diagnosis was also shorter and the proportion of deaths at 1 month was significantly lower among those with gastrointestinal presence of *H*. *capsulatum*. Subacute or chronic gastrointestinal presentations are very frequent during disseminated histoplasmosis, they seem less severe, and should lead to suspect the diagnosis in endemic areas.

## Introduction

French Guiana, a French overseas territory between Brazil and Suriname, has been afflicted by the HIV epidemic with seroprevalence exceeding 1% since the 1990s. Among patients with advanced HIV, disseminated histoplasmosis has been the most frequent AIDS-defining infection and the first cause of death.[1,2] Realization of this fact started with the dermatologists and then, with the implementation of fungal culture, spread to all physicians accelerating the identification of the fungal pathogen from a variety of samples where *H*. *capsulatum* has disseminated.[3,4] Hence, tissue samples from the skin, oral mucosa, bone marrow, lymphnodes, liver, and, very frequently, the gastrointestinal system, were examined and cultured in order to identify *H*. *capsulatum*. Although histoplasmosis is often presented as a counterpoint to tuberculosis [5,6], its main differential diagnosis, it is often a disease with a non-specific and proteiform presentation.[7,8] One of the common presentations, albeit less well known, is a presentation with febrile gastrointestinal signs and symptoms. Several authors have described cases and mention the frequency and polymorphism of such presentations.[9–14]

In this context, we aimed to describe the frequency and manifestations of gastrointestinal histoplasmosis in French Guiana, and to compare disseminated histoplasmosis cases with or without gastrointestinal involvement.

## Methods

### Ethics statement

The 1992 Histoplasmosis and HIV anonymized database was approved by the French National Institute of Health and Medical Research institutional review board (CEEI INSERM) (IRB0000388, FWA00005831 18/05/2010), by the Comité Consultatif pour le Traitement de l’Information pour la Recherche en Santé(CCTIRS) (N° 10.175bis, 10/06/2010), and the Commission Nationale Informatique et Libertés (CNIL) (n° JZU0048856X, 07/16/2010).

### Study design

A multicentric retrospective, observational study included patients between January 1, 1981 and October 1, 2014.

### Study population

Co-infections with HIV and histoplasmosis were enrolled in the Histoplasmosis and HIV database of French Guiana. Diagnosis of histoplasmosis in French Guiana rests on direct examination, fungal culture, and pathology (Gomori-Grocott and/or Periodic-Acid-Schiff (PAS) stain). There were no available *Histoplasma* antigen detection tests in French Guiana during the study period. Inclusion criteria were: age >18 years, confirmed HIV infection; first proven episode of histoplasmosis following the EORTC/MSG criteria [15]. Suspected but unproven histoplasmosis (successful empirical antifungal therapy), diagnosis only based a positive PCR, or recurrent histoplasmosis were not included.

### Study conduct

The database on histoplasmosis in HIV-infected patients was created in 1992. Cases of incident HIV-associated histoplasmosis were included in the three hospitals of French Guiana. Sociodemographic, clinical, biological, immunovirological and therapeutic data were collected on a standardized paper form until October 2014: sex, age, place of birth, symptoms on admission, clinical entrance examination, immunovirological assessment, medical imaging, mycology, pathology, treatment received, duration, dosage, route of administration. Survival data was collected for the study period.

### Statistical analysis

STATA (College Station, Texas, USA) was used for statistical analysis. For qualitative variables chi2 or Fisher tests were computed comparing the proportions between those with and without gastrointestinal involvement. Quantitative variables were described using medians and interquartile ranges, they were compared between groups with or without gastrointestinal involvement and positive gastrointestinal identification of *H*. *capsulatum* using ranksum non-parametric tests or Student’s t-test where appropriate. Variables that significantly differed between groups were included in a multivariate logistic regression model to obtain adjusted odds ratios. The model fit was tested using the Hosmer Lemeshow goodness of fit test. Statistical significance was set at P<0.05.

## Results

### Gastrointestinal symptoms and signs

Among 349 cases of disseminated histoplasmosis between January 1, 1981 and October 1, 2014, 245 (70%) had a gastrointestinal presentation. When excluding patients with concomitant opportunistic infections the proportion remained the same (150/212, (70.7%)). Among patients with concomitant opportunistic infections and digestive signs, the following infections were observed: esophageal candidiasis (22), cytomegalovirus infection (16), bacteriemia (14), cerebral toxoplasmosis (12), tuberculosis (11), chronic herpes (11), salmonellosis (6), atypical mycobacteriosis (5), cryptosporidiosis (3), cryptococcosis (2), pneumocystosis (1). Table 1 compares patients with and without gastrointestinal manifestations and positive identification of *H*. *capsulatum*.

**Table 1:** HIV-associated disseminated histoplasmosis: comparison between patients with and without gastrointestinal symptoms and signs and positive identification of H. capsulatum in the digestive tract.

Patient features	Disseminated histoplasmosis with a gastrointestinal presentation N = 65	Disseminated histoplasmosis without a gastrointestinal presentation N = 103	P
Sociodemographic			
Mean age [SD] in years	41.5 [9.2]	40.9 [10.6]	0.7
Sex M F	41 (38.3) 24 (39.3)	66 (61.7) 37 (60.7)	0.9
Geographic origin N(%)			0.001
French Guiana	19 (44.2)	24 (55.8)	
Haiti	5 (10.9)	41 (89.1)	
Suriname	14 (48.3)	15 (50.7)	
Brazil	20 (71.4)	8 (28.6)	
Guyana	2 (20)	8 (80)	
Mainland France	1 (16.7)	5 (83.3)	
Other	4 (66.6)	2 (33.3)	
Median number of months in French Guiana [IQR]	300 [96–432]	228 [96–384]	P = 0.34
Inclusion center N(%)			P = 0.11
Cayenne	39 (33.6)	77 (66.4)	
Kourou	8 (44.4)	10 (55.6)	
Saint Laurent	18 (52.9)	16 (47.1)	
HIV infection data			
Transmission mode			P = 0.4
heterosexual	62 (70.9)	93 (29.1)	
Homosexual	0 (57.1)	3 (46.9)	
IV drug use	0 (0)	3 (100)	
Transfusion	1	0	
unknown	2 (85.7)	2 (14.3)	
HAART on admission Yes No	11 (50) 55 (37.2)	10 (50) 93 (62.8)	P = 0.2
Primary prophylaxis on admission Yes No	12 (57.1) 53 (36)	9 (42.9) 94 (64)	0.06
Histoplasmosis is AIDS-classifying event Yes No	56 (43.7) 9 (22.5)	72 (56.3) 31 (77.5)	P = 0.01
History of opportunistic infection Yes No	11 (26.2) 31 (42.9)	31 (73.8) 72 (57.1)	P = 0.05
Concomitant opportunistic infection Yes No	25 (37.9) 40 (39.2)	41 (62.1) 62 (60.8)	P = 0.8
Median CD4 count on admission [IQR]			
Immunosuppression stage			P = 0.15
CD4≥200 per mm3	4 (26.6)	11 (73.4)	
CD4[50–200 [per mm3	25 (51)	24 (49)	
CD4<50 per mm3	35 (34.3)	67 (65.7)	
Clinical and paraclinical examination on admission			
Impaired WHO general performance score (>2) Yes No	24 (36.9) 41 (40)	41 (63.1) 62 (60)	P = 0.7
Fever Yes No	51 (36.4) 14 (50)	89 (63.6) 14 (50)	0.17
Weight loss Yes No	24 (39.3) 5 (50)	37 (60.7) 5 (50)	0.5
Respiratory signs Yes No	15 (21.4) 50 (51)	55 (78.6) 48 (49)	P<0.001
Superficial adenopathies >2cm Yes No	5 (14.7) 22 (46.8)	29 (85.3) 25 (53.2)	P = 0.002
Cutaneous signs Yes No	2 (15.4) 63 (40.6)	11 (84.6) 92 (59.4)	0.07
Oral signs Yes No	1 (7.1) 64 (41.6)	13 (92.9) 90 (58.4)	P = 0.01
Neurological signs Yes No	12 (40) 53 (384)	18 (60) 85 (61.6)	P = 0.8
Abdominal ultrasound performed Yes No	43 (39.1) 22 (37.9)	67 (60.9) 36 (62.1)	P = 0.8
Endoscopy performed Yes No	63 (76.8) 2 (2.3)	19 (23.2) 84 (97.7)	P<0.001
Bone marrow aspiration Yes No	13 (41.9) 52 (38)	61 (58.1) 41 (62)	P = 0.6
Standard biological tests on admission			
Hemoglobin Mean [SD]	9.5 [2]	9.3 [2.1]	P = 0.4
Neutrophils (/mm3) Median [IQR]	1550 [4–2650]	1815 [1100–2790]	P = 0.07
Platelets Median [IQR]	154500 [353–269500]	16500 [73000–249000]	P = 0.5
Serum creatinine (μmol/L) Median [IQR]	76 [60–97]	79 [66–91]	P = 0.4
Protein (g/L) Mean [SD]	81.9 [14]	80.6 [12.2]	P = 0.6
Aspartate aminotransferase (IU/L) Median [IQR]	40 [25–53]	53 [32–95]	P = 0.004
Alanine aminotransferase (IU/L) Median [IQR]	22 [15–34]	28 [17–52]	P = 0.07
Gamma glutamyl transferase (IU/L) Median [IQR]	74 [58–240]	80 [43–195]	P = 0.4
Alkaline phosphatase (IU/L) Median [IQR]	125 [87–250]	109 [71–228]	P = 0.22
Lactico Deshydrogenase (LDH)(IU/L) Median [IQR]	307 [227–407]	482 [284–1308]	P = 0.0001
CRP (mg/L) Median [IQR]	43 [15–91]	46 [13–87]	P = 0.8
Ferritin (μg/L) Median [IQR]	847 [272–1430]	1057 [660–3080]	P = 0.03
Prothrombin time Median [IQR]	76 [68–85]	86 [77–92]	P = 0.004
Diagnosis and treatment of histoplasmosis			
Delay between symptoms onset and diagnosis (days) Median[IQR]	40 [20–133]	78 [25–231]	P = 0.03
Diagnostic method			
Direct examination	36/65	50/103	P = 0.38
Fungal culture	45/65	60/103	P = 0.15
Pathology	46/65	38/103	P<0.001
Initial treatment			
Deoxycholate amphotericin B	5/65	14/99	P = 0.2
Liposomal amphotericin B	17.65	23/99	P = 0.5
Itraconazole	55.65	49/69	P = 0.2
Death at 1 month after antifungal initiation Yes No	3 (4.6) 62 (95.4)	19 (18.5) 84 (81.5)	P = 0.01

Table 1 shows there were significantly fewer deaths at one month in patients with a digestive presentation and positive identification of *H*. *capsulatum* than in patients without a gastrointestinal presentation. Patients from Brazil seemed more likely to have a digestive presentation while those from Haiti were less likely. After adjusting for potential confounding, the presence of lymphadenopathies >2cm (AOR = 0.2, IC95 = 0.04–0.7, P = 0.01), Haitian origin (AOR = 0.04, IC95 = 0.004–0.4, P = 0.006) were associated with a lower prevalence of gastrointestinal signs and positive gastrointestinal presence of *H*. *capsulatum*. Patients with a positive identification from the gastrointestinal tract were less likely to have respiratory signs, oral signs, and superficial adenopathies. Histoplasmosis was more likely to be the AIDS-classifying cause among those with gastrointestinal identification. Biologically, those with gastrointestinal *H*. *capsulatum* had lower TGO, LDH, Ferritin, prothrombin time than those without digestive signs. They had a shorter delay between symptoms onset and diagnosis and were more likely to be diagnosed through pathology than those without gastrointestinal manifestations. The duration of the study was categorized in to 4 periods: <1998, 1998–2003, 2004–2009, 2010–2014. There was a linear trend for the odds of having gastrointestinal signs and symptoms which increased significantly with time (P<0.0001). There was no such temporal trend for gastrointestinal endoscopies but there was a significant trend for an increase in the proportion of patients benefitting from and abdominal CT-scan, a gastrointestinal biopsy, and a decrease in abdominal ultrasonography.

Table 2 shows the main presentations among the 245 cases of histoplasmosis with gastrointestinal symptoms and signs, among those without any other opportunistic infections, and among those with a positive diagnosis on a gastrointestinal sample.

**Table 2:** Gastrointestinal clinical manifestations in HIV-associated disseminated histoplasmosis: nature and prevalence.

Variable	n/N all patients with gastrointestinal signs and symptoms (%)	n/N patients without any other concomitant opportunistic infection (%)	n/N patients with a positive diagnosis on a gastrointestinal sample (%)
Abdominal pain	124/245 (50.6)	76/150 (50.7)	48/65 (73.8)
Diarrhea	123/245 (50.2)	76/150 (50.7)	46/65 (70.8)
mucoid-bloody /watery diarrhea	21/122 (17.2)	11/65 (14.5)	8/37 (17.8)
Liver enlargement	98/245 (40.0)	62/150 (41.3)	16/65 (24.6)
Splenomegaly	56/245 (22.8)	34/150 (22.7)	7/65 (10.8)
Anal lesions	9/245 (3.6)	7/150 (4.7)	2/65 (3.1)
Digestive complications	24/245 (9.8)	16/150 (10.7)	10/65 (15.4)
Ascites	9/245 (3.7)	7/150 (4.7)	3/65 (4.6)
Hemorrhage	7/24 (2.9)	5/150 (3.2)	3/65 (4.6)
Dysphagia	3/245 (1.2)	2/150 (1.3)	0/65 (0)
Subocclusion	2/245 (0.8)	2/150 (1.3)	2/65 (3.1)
Perforation	1/245 (0.4)	0/150 (0)	1/65 (1.5)
Vomiting+++	1/245 (0.4)	0/150 (0)	0/65 (0)
Appendixectomy+right colonectomy	1/245 (0.4)	0/150 (0)	1/65 (1.5)
Peritonitis	1/245 (0.4)	0/150 (0)	1/65 (1.5)
Abdominal mass	1/245 (0.4)	1/150 (0.7)	1/65 (1.5)

Table 3 shows the main paraclinical findings among the 245 cases of histoplasmosis with gastrointestinal symptoms and signs and 65 patients with gastrointestinal signs and symptoms and a positive identification of *H*. *capsulatum*.

**Table 3:** Paraclinical explorations of patients with a gastrointestinal presentation, and in those with the identification of gastrointestinal *H*. *capsulatum*.

Variable	n/N with clinical signs (%)	n/N with gastrointestinal identification of *H*. *capsulatum* (%)
**Abdominal ultrasonography**	167/245 (71.3)	43/65 (66.1)
Abnormal	122/167 (72.4)	29/43 (67.4)
Hepatomegaly	83/122 (68)	19/29 (65.5)
Splenomegaly	52/122 (42.6)	10/29 (34.5)
Deep adenopathies	72/122 (58.2)	18/29 (62.1)
*Abdominal*	65/72 (90.3)	14/19 (73.7)
*Celiomesenteric*	22/72 (40.3)	8/19 (42.1)
*interaortico-caval*	16/72 (22.2)	5/19 (26.3)
*hepatic hilus*	12/72 (16.7)	2/19 (10.5)
*Lombo-aortic*	10/72 (13.9)	2/19 (10.5)
*Illiac*	4/72 (5.5)	2/19 (10.5)
*Latero-caval*	4/72 (5.5)	1/19 (5.2)
Ascites	12/122 (9.8)	2/29 (6.9)
Kidney involvement	7/122 (5.7)	2/29 (6.9)
**Abdominal CT-scanner**	75/244 (30.7)	28/65 (43.1)
**Abnormal**	63/75 (84)	25/28 (89.3)
*Hepatomegaly*	28/63 (44.4)	9/25 (36)
*Splenomegaly*	28/63 (44.4)	7/25 (28)
Deep adenopathies	47/63 (74.6)	22/25 (84)
*Abdominal*	30/48 (62.5)	15/22 (68.2)
*Celiomesenteric*	30/48 (62.5)	15/22 (68.2)
*Retroperitoneal*	16/48 (33.3)	5/22 (22.7)
*Lombo-aortic*	10/48 (20.8)	2/22 (9.1)
*interaortico-caval*	9/48 (18.7)	6/22 (27.3)
*Illiac*	8/48 (16.7)	2/22 (9.1)
*Hepatic hilus*	4/48 (8.3)	2/22 (9.1)
*Latero-caval*	1/48 (2)	0/22 (0)
Ascitis	13/63 (20.6)	1/25 (4)
**Digestive endoscopy**	117/245 (47.7)	63/65
*Upper digestive tract endoscopy*	70/117 (59.8)	33/63 (52.4)
**Abnormal**	39/70 (54.3)	19/33 (57.6)
Location		
*Stomach*	13/39 (33.3)	9/19 (47.4)
*Esophagus*	10/39 (25.6)	5/19 (26.3)
*Duodenum*	7/39 (17.9)	6/19 (31.6)
*aerodigestive tract bifurcation*	1/39 (2.5)	1/19 (5.3)
Aspect (by descending frequency)		
*Associated Esophageal candidiasis*	18/39 (46.1)	5/19 (26.3)
*Ulcerations*	13/39 (33.3)	9/19 (47.4)
*Non specific inflammation*	9/39 (23)	6/19 (31.6)
*Erosions*	2/39 (5.1)	1/19 (5.3)
*Ulcerovegetating lesions*	2/39 (5.1)	1/19 (5.3)
*Lower digestive tract endoscopy*	77/117 (65.8)	57/63 (90.5)
**Abnormal**	59/77 (76.6)	53/57 (93)
Location		
*Transverse colon*	34/59 (57.6)	33/53 (62.3)
*Right colon*	30/59 (50.8)	29/53 (54.7)
*Caecum*	29/59 (49.1)	28/53 (52.8)
*Left colon*	27/59 (45.7)	25/53 (47.2)
*Sigmoid*	17/59 (28.8)	15/53 (28.3)
*Rectum*	16/59 (27.1)	12/53 (22.6)
*Distal ileon*	6/59 (10.2)	6/53 (11.3)
*Anus*	5/58 (8.6)	3/53 (5.8)
Aspect		
*Ulcerations*	49/59 (83)	46/53 (86.8)
*Non specific inflammation*	12/59 (20.3)	10/53 (18.9)
*Necrotic ulcerations*	2/59 (13.5)	2/53 (3.8)
*Ulcerovegetating lesions*	5/59 (8.5)	5/53 (9.4)
*Stenosis*	3/59 (5)	2/53 (3.8)
*Erosions*	2/59 (3.4)	33/53 (3.8)

Half of patients with gastrointestinal signs had abdominal pain or diarrhea, mostly watery. This proportion was even greater among those with a positive diagnosis on a digestive sample (Table 2). Half of patients with abdominal pain had diarrhea (63/124) and half of those with diarrhea (63/123) had abdominal pain. Among those with diarrhea, 84% had colonoscopy and among those with abdominal pain 68.5% had a colonoscopy. A significant proportion of patients also had hepatomegaly and, to a lesser degree, splenomegaly. Patients with abdominal pain were less likely to have hepato or splenomegaly (OR = 0.3, 95%CI = 0.2–0.6, P<0.001) and those with diarrhea were also less likely to have hepato or splenomegaly (OR = 0.3, 95%CI = 0.2–0.6, P<0.001).

The most frequently prescribed procedure was by far abdominal ultrasonography (71%), far more prescribed than gastrointestinal endoscopy (47.7%) and abdominal CT-Scan (30.7%) (Table 3). However, most patients with diarrhea (80%) or abdominal pain (68.5%) benefitted from colonoscopy. Ultrasonography and CT-Scanner were usually abnormal in 72–80% of the cases, whereas endoscopy was abnormal in 54% of cases. Medical imagery showed deep adenopathies in 58% of ultrasounds, and 74% of CT-Scanners, mostly abdominal and celiomesenteric. Ascites was observed in 10% of ultrasounds and 20% of CT-Scans. Endoscopy was more frequently abnormal in the lower than in the upper digestive tract. Although the whole colon could be involved, the right and transverse colon were the most frequent lesional sites. The most frequent lesional aspects were ulcerations and non-specific inflammation, for both the upper digestive tract and lower digestive tract.

Overall, among those having benefitted from gastrointestinal biopsies 65/117 (55%) had a positive identification of Histoplasma on the sample; 32 (27.3%) by direct examination, 44 (37.6) by culture, and 45 (38.4%) by pathology. Biopsies performed during colonoscopy were more contributive than those performed during oesogastroduodenoscopy whether considering direct examination (respectively 56.4% vs 21.7% positive), fungal culture (respectively 74.1% vs 40.9% positive), or pathology (respectively 72.9% vs 28.6% positive).

Those with mucoid/bloody diarrhea were more likely to have gastrointestinal complications (OR = 5.1, 95%CI = 1.3–18.1, P = 0.002), and anal lesions (OR = 8.2, 95%CI = 0.9–102, Fisher exact P = 0.02).

Among those with an abnormal abdominal ultrasonography 44% had a single anomaly, 37% had 2 anomalies and 18% had 3 distinct anomalies of the upper digestive tract. Among those with an abnormal upper digestive tract endoscopy 81% had single lesions, and 11% had 2 distinct lesions. Among those with an abnormal lower digestive tract endoscopy 59% had single lesions, and 8% had 2 distinct lesions, 27% had between 3 and 10 distinct lesions, and 5% had more than 10 distinct lesions of the lower digestive tract. Among those with an abnormal abdominal scanner 43% had abnormalities, and 35% had 2 distinct abnormalities, and 22% had had 3 or more abnormalities. Among lesions visible on the abdominal CT-scanner colonic lesions were the most frequent with thickening of the colonic wall (N = 7) and 2 tumoral masses. In three cases the kidney was involved and in 1 patient the pancreas.

## Discussion

The present study emphasizes the high prevalence of gastrointestinal signs during disseminated histoplasmosis. The most frequent signs were abdominal pain, diarrhea, and hepatomegaly. Diarrhea was mostly watery. Rarely, some patients presented with pseudo-occlusive syndromes, with tumor-like aspects, perforations and peritonitis, or severe hemorrhage. These observations are concordant with published case series. [11,13,14,16,17] Over time the prevalence of gastrointestinal manifestations seemed to increase. This is perhaps linked to a greater awareness of the overall presence of disseminated histoplasmosis, and the increase in the use of abdominal CT-Scan and gastrointestinal biopsies to explore patients.

When comparing disseminated histoplasmosis with gastrointestinal signs and a positive identification of *H*. *capsulatum* with disseminated histoplasmosis without gastrointestinal signs and symptoms, patients with adenopathies >2cm, than those without adenopathies >2cm. Patients with gastrointestinal signs and symptoms and a positive identification of *H*. *capsulatum* were more likely to have a decreased prothrombin time, had lower Ferritin, TGO and LDH concentrations–often correlates of severity—than those without a gastrointestinal presentation. The interval between symptom’s onset and diagnosis was shorter and the risk of death at one month was lower in those with gastrointestinal symptoms and a positive identification of *H*. *capsulatum* than in those without gastrointestinal symptoms. A possibility would be that a quicker diagnosis through endoscopy eventually explained a more limited dissemination and a lower case-fatality. An intriguing finding, which remained significant after multivariate analysis, was that Haitians seemed less likely to have gastrointestinal presentations than South American populations. This could just be random associations, but one could discuss differences in microbiota, endemic gastrointestinal pathogens, or cultural feeding practices that somehow modulate the risk of gastrointestinal localizations. Inflammatory foci may attract infected monocytes/macrophages that hence bring the fungus to the digestive tube’s rich immunological structures. Finally, although it is considered that intestinal lesions in histoplasmosis generally follow a hematogenous fungus dissemination experimental studies in Hamsters suggested the intestinal tract was a possible portal of entrance of the infection, albeit not a major one.[18] Hence an intriguing hypothesis would be that among immunocompromised patients, perhaps those living in areas where enteric parasites and bacteria are common, some patients with disseminated histoplasmosis and gastrointestinal manifestations acquired the fungus through the intestinal tract. The present study’s limitations are linked to its retrospective nature. All patients with disseminated histoplasmosis were explored differently according to their clinical presentation. Hence, all did not have endoscopies and digestive biopsies, all did not benefit from a CT scan, diagnosis was confirmed in different ways at different sites … all of which are possible biases. Nevertheless, to our knowledge this is the largest published cohort describing real life data focused on a frequent presentation of a major opportunistic infection.

In conclusion, gastrointestinal signs and symptoms were very frequent in patients with disseminated histoplasmosis. Whereas imagery can identify lesions and adenopathies, endoscopies, notably colonoscopy, allow biopsies and opportunities to identify the pathogen either through mycological methods or histopathology. It is important for clinicians in endemic areas to suspect disseminated histoplasmosis and prescribe endoscopies when encountering gastrointestinal symptoms in a patient with advanced HIV. A quicker diagnosis–through endoscopy—may explain why we observed that severity and case fatality was lower in those with gastrointestinal identification of *H*. *capsulatum* than in those without gastrointestinal symptoms.
